# The first record of *Propomacrusbimucronatus* (Pallas, 1781) (Coleoptera, Scarabaeidae) from Iraq, with notes on its distribution and phenology in the Near East

**DOI:** 10.3897/BDJ.10.e96601

**Published:** 2022-12-30

**Authors:** Hani Ahmed Ibrahim, Ariel-Leib-Leonid Friedman, Avand Faesal Fayq

**Affiliations:** 1 Horticulture Department, Technical College of Akre, Duhok Polytechnic University, Kurdistan Region, Akre, Iraq Horticulture Department, Technical College of Akre, Duhok Polytechnic University, Kurdistan Region Akre Iraq; 2 The Steinhardt Museum of Natural History and Israel National Center for Biodiversity Studies, Tel Aviv, Israel The Steinhardt Museum of Natural History and Israel National Center for Biodiversity Studies Tel Aviv Israel; 3 Directorate of Education - Akre / General Directorate of Education - Duhok. Kurdistan Region, Akre, Iraq Directorate of Education - Akre / General Directorate of Education - Duhok. Kurdistan Region Akre Iraq

**Keywords:** *Propomacrusbimucronatus*, Scarabaeidae, Euchirini, saproxylic, new record, zoogeography, phenology

## Abstract

**Background:**

*Propomacrusbimucronatus* (Pallas, 1781), the Mediterranean long-armed scarab, is a large saproxylic beetle, occurring in the east Mediterranean and south-east Europe, sparse throughout its entire distributional range, often considered as rare, threatened or extinct species.

**New information:**

*Propomacrusbimucronatus* is recorded for the first time from Kurdistan, Iraq. The new data on its distribution and phenology in Iraq and in Israel is published for the first time, compared with the previously-published data and analysed.

## Introduction

The tribe Euchirini, sometimes considered as a separate subfamily Euchirinae (Young 1989, Smith et al. 2006, Muramoto 2012, Šípek et al. 2011, Bezděk 2016), literally long-armed scarabs, as they are sometimes referred to, due to the extremely long fore legs of the males, is a small group of large beetles (28-80 mm long), occurring in tropical and subtropical Asia, Middle East and south-east Europe. The occurrence of Euchirini is usually associated with densely forested highlands covered with old-growth broad-leaved trees, alluvial forests and growths around small streams and rivers, all with an abundance of trees with holes required for the survival of both immature stages and adults. The species for which the biology is known are breeding in the rotten wood of old trees (Arrow 1917, Young 1989, Šípek et al. 2011). Euchirini comprises 15 species in three genera, *Cheirotonus* Hope, 1841 (10 spp.), *Euchirus* Burmeister and Schaum, 1840 (2 spp.) and *Propomacrus* Newman, 1837 (3 spp.), while only *Cheironotus*, with nine species and *Propomacrus* are recorded for the Palaearctic Region (Arrow 1917, Young 1989, Muramoto 2012, Bezděk 2016

The genus *Propomacrus* comprises two species in the Oriental Region and eastern parts of the Palaearctic Region (China, Bhutan, Nepal) and two species, *P.bimucronatus* (Pallas, 1781) and *P.cypriacus* Alexis and Makris, 2002 in the West Palaearctic realm (Young 1989, Muramoto 2012, Bezděk 2016). However, the status of *P.cypriacus* is debatable: Alexis and Makris (2002) consider it as an endemic of Cyprus, while Sfenthourakis et al. (2017) suggest that it is a subspecies of the widely-distributed *P.bimucronatus* (Sfenthourakis et al. 2017).

*Propomacrusbimucronatus* (Pallas, 1781) was described from Turkey and recently is recorded from Macedonia, Bulgaria, Greece, Turkey, Syria, Lebanon, Israel and Iran (Young 1989, Muramoto 2012, Bezděk 2016), but is uncommon throughout the entire area of its distribution. It is listed under the category NT (near threatened) (European Commission, Directorate-General for Environment et al. 2010, Nieto et al. 2010).

Larvae of *P.bimucronatus* develop in the rotten wood of old mature trees with internal cavities. They had been recorded from *Quercus* L. (e.g. *Quercuscalliprinos* Webb, *Q.cerris* L., *Q.ithaburensis* Decne., *Quercus* sp.) (Arrow 1917, Young 1989, Šípek et al. 2011, Sabatinelli 2013, Buse et al. 2013), *Platanusorientalis* L. (Lumaret and Tauzin 1992, Šípek et al. 2011, Sfenthourakis et al. 2017), *Ceratoniasiliqua* L. (Sfenthourakis et al. 2017), *Alnusorientalis* Decne. (Sfenthourakis et al. 2017) and *Prunusdulcis* Batsch (Sfenthourakis et al. 2017). It is most probable that its host range is wider, for example, 27 adults were trapped in chestnut (*Castaneasativa* L.) plantations, although attracted to traps baited with boiled grape juice (Onucar and Ulu 1986). Adults are attracted to juicy fruit, for example, *Cordiamyxa* L.(Arrow 1917, Young 1989).

## Materials and methods

The adult males and females of *P.bimucronatus* were collected in July-August in the different horticultural areas of Kurdistan Province, Iraq, namely in Akre and Amadiya, during the survey of insects. The beetles were collected by hand, while attracted to light, then the specimens were transferred to the laboratory and put into a freezer. The studied material from Iraq is deposited in the Horticulture Department, Technical College - Akre / Duhok Polytechnic University, Kurdistan, Iraq (HDTAI). The studied material from Israel is deposited in the National Collection of Insects, the Steinhardt Museum of Natural History, National Research Center, Tel Aviv University, Israel (SMNHTAU) and private collections of J. Buse, Black Forest National Park, Freudenstadt, Germany (cBU) and E. Rössner, Schwerin, Germany (cRO).

Transliterated names of the localities in Iraq follow Google Maps.

Transliterated names of localities in Israel follow the Israel Touring Map and List of settlements (Anonymous 2009). Regional subdivision of Israel (Table 1) follows Theodor (1975), with changes made by Friedman (2018).

## Taxon treatments

### Propomacrus bimucronatus

(Pallas, 1781)

8D407939-E381-5813-8EC8-642E1F537694

Tribe Euchirini Hope 1840
Genus *Propomacrus* Newman, 1837: 255 - Newman (1837)
(type species *Propomacrusarbaces* Newman, 1837 (= *Scarabaeusbimucronatus* Pallas, 1781))
*Propomacrusbimucronatus* Pallas, 1781: 13 (*Scarabaeus*) - Pallas (1781)
jun. syn. *Propomacrusarbaces* Newman, 1837: 256

#### Materials

- **Type status:** Other material. **Occurrence:** sex: 1 male, 2 females; preparations: pinned; occurrenceID: 57C89313-7C04-5723-9C96-13BF1082CB26; **Location:** country: Iraq; stateProvince: Kurdistan; locality: Akre; verbatimCoordinates: 36.758036, 43.900535; **Identification:** identifiedBy: A.L.L. Friedman; **Event:** verbatimEventDate: 6.vii.2022; **Record Level:** ownerInstitutionCode: HDTAI
- **Type status:** Other material. **Occurrence:** sex: 1 female; preparations: pinned; occurrenceID: 825AF48F-2F59-52E0-9D4B-D7DE6D64701A; **Location:** country: Iraq; stateProvince: Kurdistan; locality: Amadiya District, Mangesh, Zewki Kandal village; verbatimCoordinates: 37.0215515 43.0269128; **Identification:** identifiedBy: A.L.L. Friedman; **Event:** samplingProtocol: house lights; verbatimEventDate: 24.viii.2022; habitat: almond orchard; **Record Level:** ownerInstitutionCode: HDTAI
- **Type status:** Other material. **Occurrence:** sex: 2 females; preparations: pinned; occurrenceID: 0374D62B-E567-5E4D-B715-58FB1A7070BB; **Location:** country: Israel; locality: Newe Ativ; **Identification:** identifiedBy: A.L.L. Friedman; **Event:** verbatimEventDate: 26-29.viii.1981; **Record Level:** ownerInstitutionCode: SMNHTAU
- **Type status:** Other material. **Occurrence:** sex: 1 female; occurrenceID: EA66FFE0-63A9-521F-9533-5FB703573100; **Location:** country: Israel; locality: Panyas; **Identification:** identifiedBy: A.L.L. Friedman; **Event:** samplingProtocol: light trap; verbatimEventDate: 30.vii.2002; **Record Level:** ownerInstitutionCode: SMNHTAU
- **Type status:** Other material. **Occurrence:** sex: 1 male; occurrenceID: 53C17C19-0EA1-5B13-B550-0AE1F6331777; **Location:** country: Israel; locality: Dan; **Identification:** identifiedBy: A.L.L. Friedman; **Event:** verbatimEventDate: 20.x.1966; **Record Level:** ownerInstitutionCode: SMNHTAU
- **Type status:** Other material. **Occurrence:** sex: 1 female; occurrenceID: 316A5773-E0AD-599C-BA63-633E8482EDF8; **Location:** country: Israel; locality: Dan; **Identification:** identifiedBy: A.L.L. Friedma; **Event:** verbatimEventDate: ix.1981; **Record Level:** ownerInstitutionCode: SMNHTAU
- **Type status:** Other material. **Occurrence:** sex: 1 female; occurrenceID: 9D54ED3C-F10F-5408-B7FB-57E239AC6C39; **Location:** country: Israel; locality: Hermon Field School; **Identification:** identifiedBy: A.L.L. Friedman; **Event:** verbatimEventDate: viii.1992; **Record Level:** ownerInstitutionCode: SMNHTAU
- **Type status:** Other material. **Occurrence:** sex: 1 female; occurrenceID: 260A1DF9-0F7B-530C-8AA9-B3F8C45E2AB5; **Location:** country: Israel; locality: Sede Nehemya; verbatimLocality: Huliyo; **Identification:** identifiedBy: A.L.L. Friedman; **Event:** verbatimEventDate: 27.viii.1973; **Record Level:** ownerInstitutionCode: SMNHTAU
- **Type status:** Other material. **Occurrence:** sex: 1 female; occurrenceID: 49D59DB6-0255-5047-A5D8-90DDAFED13E2; **Location:** country: Israel; locality: Sede Nehemya; verbatimLocality: Huliyo; **Identification:** identifiedBy: A.L.L. Friedman; **Event:** verbatimEventDate: 13.ix.1973; **Record Level:** ownerInstitutionCode: SMNHTAU
- **Type status:** Other material. **Occurrence:** sex: 1 female; occurrenceID: 56A59831-E970-5701-8A9B-66B174DBF50A; **Location:** country: Israel; locality: Senir; **Identification:** identifiedBy: A.L.L. Friedman; **Event:** verbatimEventDate: 22.viii.1969; **Record Level:** ownerInstitutionCode: SMNHTAU
- **Type status:** Other material. **Occurrence:** sex: 1 male; occurrenceID: F165E84F-D56B-5644-B8E4-BA08944B9B01; **Location:** country: Israel; locality: Nahal Keziv; **Identification:** identifiedBy: A.L.L. Friedman; **Event:** verbatimEventDate: 20.v.1999; **Record Level:** ownerInstitutionCode: SMNHTAU
- **Type status:** Other material. **Occurrence:** sex: 1 male; occurrenceID: B2F88E66-D017-5165-938C-F2BEA29229AA; **Location:** country: Israel; locality: Nahal 'Ammud; **Identification:** identifiedBy: A.L.L. Friedman; **Event:** verbatimEventDate: 23.ix.2003; **Record Level:** ownerInstitutionCode: SMNHTAU
- **Type status:** Other material. **Occurrence:** sex: 1 female; occurrenceID: 2CF21B41-CD2F-5A39-A4D4-576C47FF5218; **Location:** country: Israel; locality: Hosha'aya; **Identification:** identifiedBy: A.L.L. Friedman; **Event:** samplingProtocol: light trap; verbatimEventDate: 9.vii.2022; **Record Level:** ownerInstitutionCode: SMNHTAU
- **Type status:** Other material. **Occurrence:** sex: 1 male; occurrenceID: FAC33A72-58AF-593C-9852-72A76214166E; **Location:** country: Israel; locality: Bet Lehem haGelilit; **Identification:** identifiedBy: A.L.L. Friedman; **Event:** verbatimEventDate: 12.viii.2022; **Record Level:** ownerInstitutionCode: SMNHTAU
- **Type status:** Other material. **Occurrence:** sex: 1 female; occurrenceID: 72DC4CED-B360-5B7C-B322-8B483C6DEA8D; **Location:** country: Israel; locality: Bet Shearim; **Identification:** identifiedBy: A.L.L. Friedman; **Event:** verbatimEventDate: 16.ix.1957; **Record Level:** ownerInstitutionCode: SMNHTAU
- **Type status:** Other material. **Occurrence:** sex: 1 female; occurrenceID: 7E21ECEB-DB19-5300-970F-85AE49F69A56; **Location:** country: Israel; verbatimLocality: Haifa, Carmel; **Identification:** identifiedBy: A.L.L. Friedman; **Event:** verbatimEventDate: 6.vii.1945; **Record Level:** ownerInstitutionCode: SMNHTAU
- **Type status:** Other material. **Occurrence:** individualCount: 1; occurrenceID: 6E6F0765-DCD3-5C38-9DC7-B8DC07DD98CC; **Location:** country: Israel; locality: Carmel Ridge, Horeshat haArba’im; **Identification:** identifiedBy: E. Rössner; **Event:** samplingProtocol: window trap; eventTime: 10-31.vii.2009; **Record Level:** collectionCode: cBU, cRO
- **Type status:** Other material. **Occurrence:** individualCount: 1; occurrenceID: 5454FD4E-5909-5FB0-A635-EF89A04E44EA; **Location:** country: Israel; locality: Carmel Ridge, Horeshat haArba’im; **Identification:** identifiedBy: E. Rössner; **Event:** samplingProtocol: window trap; eventTime: 31.vii-13.viii.2009; **Record Level:** collectionCode: cBU, cRO
- **Type status:** Other material. **Occurrence:** individualCount: 4; occurrenceID: BFA8A1DD-573A-5854-A38D-7B3489CC07EB; **Location:** country: Israel; locality: Carmel Ridge, Horeshat haArba’im; **Identification:** identifiedBy: E. Rössner; **Event:** samplingProtocol: window trap; eventTime: 28.viii-10.ix.2009; **Record Level:** collectionCode: cBU, cRO
- **Type status:** Other material. **Occurrence:** sex: 1 female; occurrenceID: D2B06E51-733A-57EC-8ADB-22B5DE063C61; **Location:** country: Israel; locality: Pardes-Hanna; **Identification:** identifiedBy: A.L.L. Friedman; **Event:** verbatimEventDate: vi.1946; eventRemarks: found dead; **Record Level:** ownerInstitutionCode: SMNHTAU
- **Type status:** Other material. **Occurrence:** sex: 1 female; occurrenceID: FF2B666F-2184-56BA-8A36-29C695C6B968; **Location:** country: Israel; locality: Pardes-Hanna; verbatimLocality: Meged; **Identification:** identifiedBy: A.L.L. Friedman; **Event:** verbatimEventDate: 5.ix.1948; **Record Level:** ownerInstitutionCode: SMNHTAU
- **Type status:** Other material. **Occurrence:** sex: 1 female; occurrenceID: E58CF609-A02F-5D3A-8EEA-71AF3D36FBEE; **Location:** country: Israel; locality: Tel Aviv; **Identification:** identifiedBy: A.L.L. Friedman; **Event:** verbatimEventDate: summer 1954; **Record Level:** ownerInstitutionCode: SMNHTAU
- **Type status:** Other material. **Occurrence:** sex: 1 female; occurrenceID: 21592425-BEA4-5D31-883D-C9123A0F9A8F; **Location:** country: Israel; locality: Miqwe Israel Agricultural School; **Identification:** identifiedBy: A.L.L. Friedman; **Event:** verbatimEventDate: 23.vii.1945; **Record Level:** ownerInstitutionCode: SMNHTAU
- **Type status:** Other material. **Occurrence:** sex: 1 male; occurrenceID: AE7C2A57-D1A6-5C91-A082-E8594275E5FA; **Location:** country: Israel; locality: Sha'ar haGay; **Identification:** identifiedBy: A.L.L. Friedman; **Event:** verbatimEventDate: iv.1958; fieldNotes: f; **Record Level:** ownerInstitutionCode: SMNHTAU
- **Type status:** Other material. **Occurrence:** occurrenceID: BF9D2569-585E-5E9D-BFAF-59A83C68F642; **Location:** country: Israel; locality: Yerushalayim, Refa’im Valley; **Identification:** identifiedBy: A. Orlov; **Event:** verbatimEventDate: 11.ix.2014; fieldNotes: found dead early in the morning; **Record Level:** ownerInstitutionCode: SMNHTAU

#### Description

The species can be easily distinguished from other Scarabaeoidea in the region by the following diagnostic characters: Body length 30-45 mm. Body and appendages dark brown, shiny, glabrous, apart from the coarsely sculptured pronotum. Pronotum swollen anteriorly, laterally projected into wide pointed angles, laterally and latero-ventrally covered by short reddish, yellowish or golden hair. Fore legs of a male with femur and, in particular, tibia strongly enlarged; tibiae of both sexes possess strong mucro and spurs.

#### Distribution

North Macedonia, Bulgaria, Greece, Turkey, Syria, Lebanon, Israel, Iran and Iraq (new record).

## Analysis

**Figure 1.**
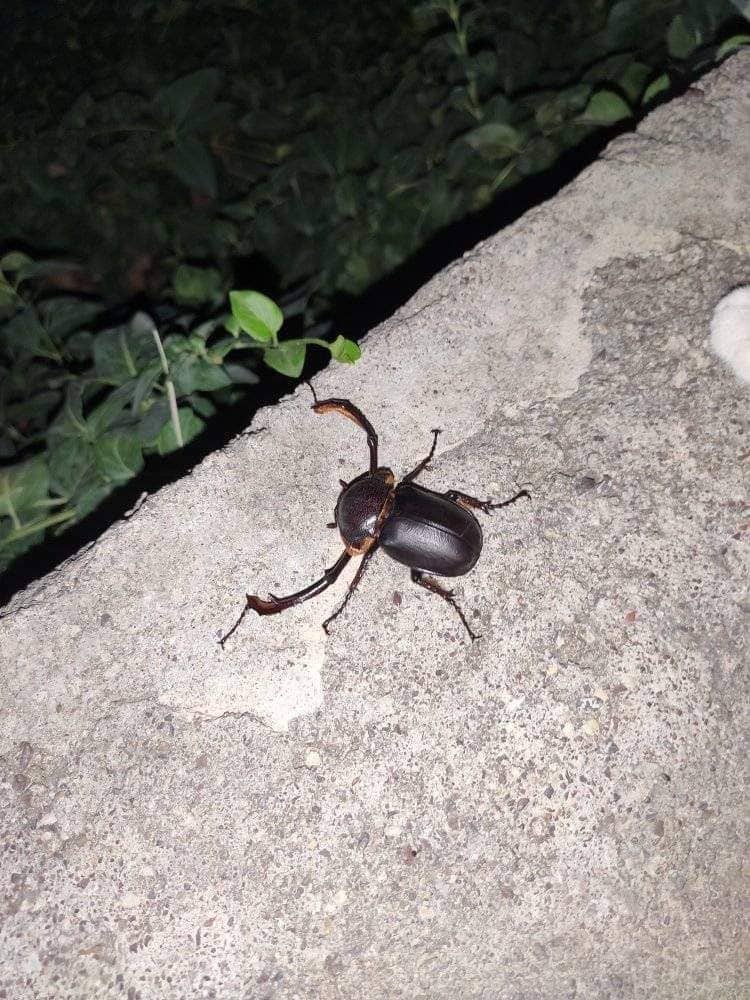
*Propomacrusbimucronatus*, male, Akre, Iraq (photograph by H. Ibrahim).

Four specimens of *P.bimucronatus* were collected in July-August 2022 in Kurdistan, Iraq (Fig. 1). The beetles were collected on the southern and south-western slopes of the mountainous ranges of northern and north-eastern Kurdistan (part of the Zagros Mountains). Three specimens were collected in Akre District (Fig. 3) and one specimen was attracted to the house lights at night in Amadiya District (Fig. 4). The beetles were collected in the outskirts of the settlements, in the areas mainly covered by planted vegetation, agricultural or ornamental. For example, in Akre, it was collected at the town edge, in a small neighbourhood surrounded by walnut trees (*Juglansregia* L.), quince (*Cydoniaoblonga* Mill.) and various other stone fruits (Rosaceae), olive (*Oleaeuropaea* L.), fig (*Ficuscarica* L.), loquat (*Eriobotryajaponica* (Thunb.) Lindl.), pines (*Pinus* spp.) and various ornamental trees, in the close proximity to the forested slopes of the mountains, covered by natural vegetation. Kurdistan is, in general, a mountainous region; the beetles were collected at 800 m (Akre) and 900 m (Amadiya) (Fig. 5).

As only adult specimens were collected, there are no data on the larval development of *P.bimucronatus* in Kurdistan. Relying on the previous records, we can assume that the larvae inhabited either old oaks or plane trees in the close proximity. The oriental plane (*Platanusorientalis* L.) is extant in Iraq, but its distribution is limited to 1000 m altitude (Barstow and Rivers 2017). Several species of oaks are recorded from Iraqi Kurdistan, for example, *Quercusaegilops* L., *Q.infectoria* Oliv., *Q.libani* G. Olivier and *Q.macranthera* Fisch. & C.A.Mey. ex Hohen. (Khwarahm 2020), any of them can be a host, if their trunk is old enough and hollow. The collecting localities of *P.bimucronatus* in Kurdistan correspond well with the distribution of *Q.aegilops*, which is considered to be the most common oak species in Kurdistan (Khwarahm 2020). We suggest that old walnut trees (*J.regia*) can also be the host of *P.bimucronatus*, but it is not more than a bare assumption, based on the existence of the old large walnut trees in one of the localities (Akre). On the other hand, the walnut, as well as quince, fig, loquat etc., can attract adult beetles by its fruit other than by its wood.

The adults' activity season in Kurdistan matches this in other areas of *P.bimucronatus* distribution in the summer: May-August recorded for Turkey (Onucar and Ulu 1986, Lumaret and Tauzin 1992) and July-September recorded for Cyprus (Sfenthourakis et al. 2017), although Ward and Neophytou (1992) recorded three specimens in April. The same phenological pattern is demonstrated by the *P.bimucronatus*' southernmost population in Israel, as we show below.

Although *P.bimucronatus* was already recorded from Israel by Bodenheimer (1937), it was never coherently surveyed and no data were published. The following data are based on the material housed in SMNHTAU (21 specimens collected in 1945-2022 throughout Israel) and six specimens collected by Jörn Buse on the Carmel Ridge in 2009 (Buse et al. 2013 J. Buse, pers. com.) (Table 1). In the past, *P.bimucronatus* was distributed throughout the Mediterranean vegetation zone of Israel: Mt. Hermon, Golan Heights, Hula Valley, Upper Galilee, Lower Galilee, Yizre'el Valley, Carmel Ridge, Coastal Plain, Foothills of Judea and Judean Hills. There was no preference to the higher altitudes, most of the earliest collected specimens being collected between the 20 and 50 m (Tel Aviv, Miqwe Yisrael, Haifa, Pardes-Hanna) to 300 m (Sha'ar haGay). All old records (1940s-1950s) are from the Coastal Plain, Carmel Ridge, Yizre'el Valley, Lower Galilee and Judean Foothills, which appears to be a bias, hence these areas were more populated already at this time. However, there are no records from these overpopulated areas with strongly developed infrastructure and agriculture after 1958. The later records (from 1966 to today) are predominantly from the northern mountainous and, therefore, more temperate parts of the country: Mt. Hermon, Golan Heights, Hula Valley, Upper and Lower Galilee, Carmel Ridge and Judean Hills or more humid areas, like the Hula Valley. The common ground of the localities in which *P.bimucronatus* was found - these are the only areas in Israel covered by the Mediterranean forest, comprises mainly oaks (*Quercus* spp.) (some of them old veteran trees) and terebinths (*Pistacia* spp.) or water edges with oriental plains (*Platanusorientalis*), growing along the water. Most of the Israeli specimens where collected in July-September; however, in the past, the adults appeared as early as April (1958) and were still active at the end of October.

## Discussion

Although Young (1989) predicted the existence of *P.bimucronatus* in Iraq, based on its occurrence in eastern Turkey and western Iran), his assumption was proved only recently, when twelve specimens were collected in July-August 2022.

It appears strange that these large conspicuous beetles were overlooked for years and found only recently. Several explanations can be suggested, although none of them is completely convincing:

1. An insufficient rate of entomological research in Kurdistan in the previous years. However, in other areas of its distribution (e.g. Israel, Cyprus, Turkey), in which the collecting activities are more intensive, *P.bimucronatus* are as well rarely and occasionally collected. It is not collected or spotted every year. The last time adults were spotted (Lower Galilee, Qiryat Tiv'on, M. Ben-Ari, pers. comm.) or collected (Yerushalayim, A. Orlov, SMNHTAU) in Israel was in 2014.

2. Nocturnal activity of adults makes it difficult to nearly impossible to find them during day-time (the specimen collected on the banks of Nahal Keziv in 1999 was collected at day-time by hand, V. Chikatunov, pers. comm.). On the other hand, *P.bimucronatus* are attracted to lights and, therefore, allegedly are easier to be collected, but obviously do not meet the expectations. For example, only two specimens were collected during 1999-2003 of the most intensive light-trapping project with 54 light traps spread throughout Israel (Chikatunov et al. 2006).

3. The populations of *P.bimucronatus* occur only in very remote areas, deep in the forests, in mountains. In this context, the collecting of a specimen in 2014 in Yerushalayim, inside the city, is remarkable. This means that a small population exists (or at least existed eight years ago) in the semi-developed park inside a large modern overpopulated city. This record correlates nicely with collecting of a specimen inside the town of Akre. In the 1940s-1950s, *P.bimucronatus* were collected in large cities like Tel Aviv and Haifa, in the Coastal Plain, only slightly above the sea level.

4. The activity of *P.bimucronatus* corresponds to the warmest and less fruitful season in the Middle East, the late summer. It can be a human factor rather than natural reasons that the beetles are collected rarely. In this habit, the phenology of *P.bimucronatus* corresponds to the phenology of the large saproxylic Cerambycidae (e.g. *Anthracocentrus*, *Aromia*, *Cerambyx*, *Mesoprionus*, *Monocladum*, *Prinobius*, *Rhaesus*) and Lucanidae (*Lucanus*, *Dorcus*) and is completely different from most of the Scarabaeidae, whose peak of activity is in spring (personal observations).

*P.bimucronatus* is often mentioned as a rare threatened species (European Commission, Directorate-General for Environment et al. 2010, Nieto et al. 2010). Reitter (1961) considered it extinct, finding no new material in European collections, which definitely is proved as incorrect. It is not clear, however, how common it is, as its activity is quite irregular. In most of the places, they appear very irregularly, once in 2-3 or more years. The previous specimen was spotted in the same area, Lower Galilee, eight years earlier (M. Ben-Ari, pers. comm.); six specimens were collected with window traps on the top of the Carmel Ridge in 2009 and none in the same place in 2008 (Buse et al. 2013).

**Figure 2.**
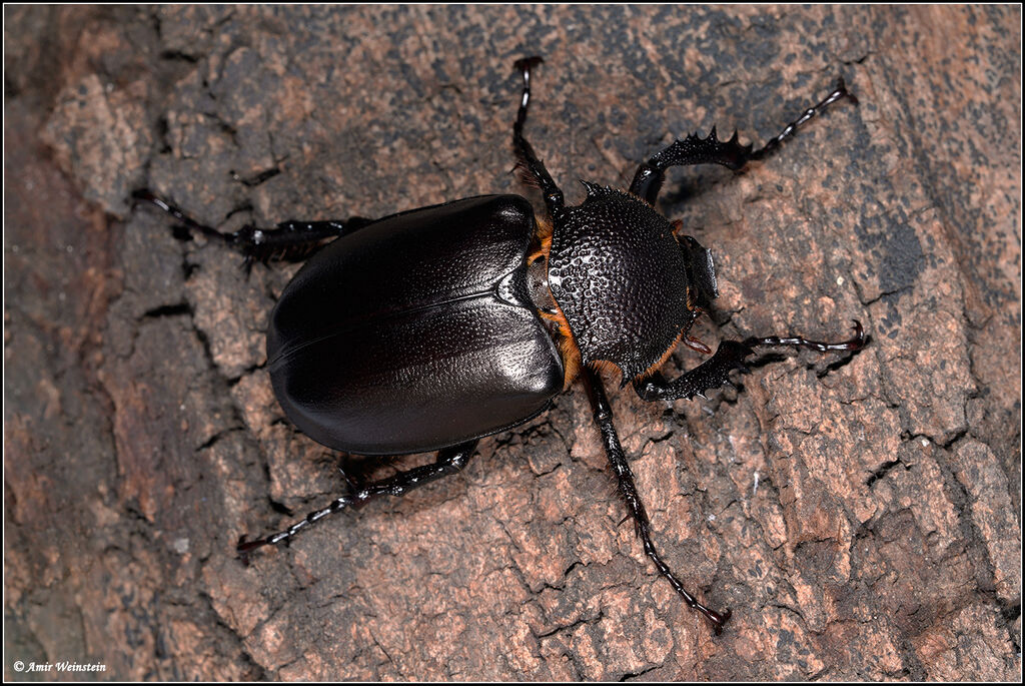
*P.bimucronatus*, female, Hosha’aya, Israel (photograph by Amir Weinstein).

**Figure 3.**
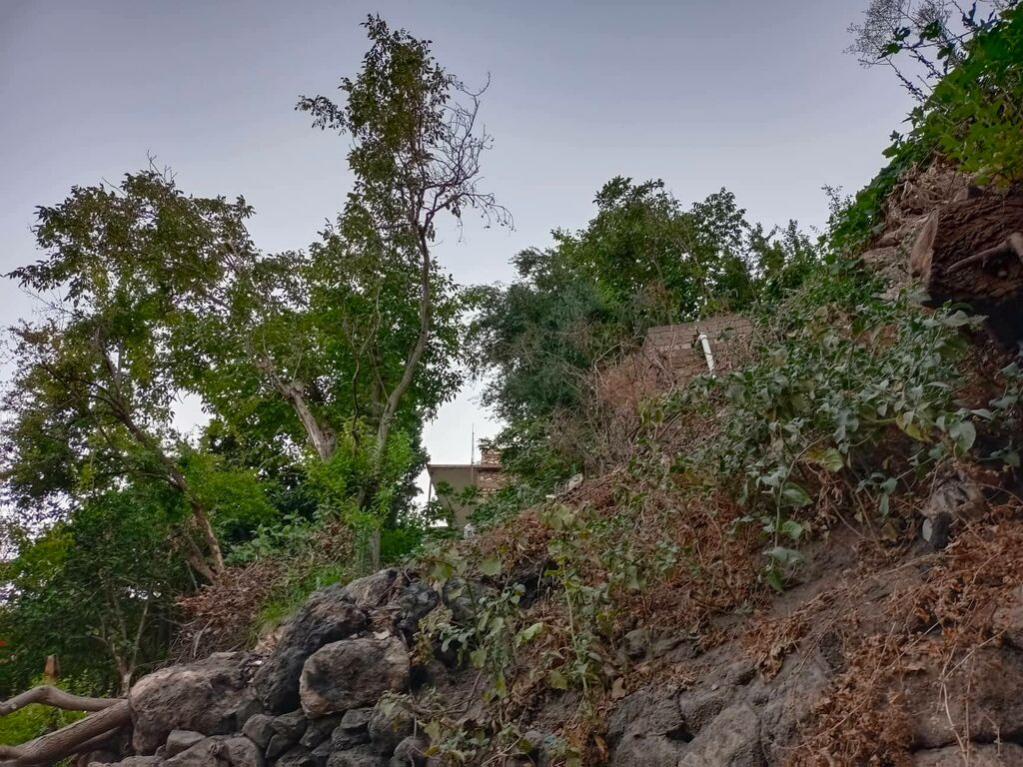
Collecting site in Akre (photograph by H. Ibrahim).

**Figure 4.**
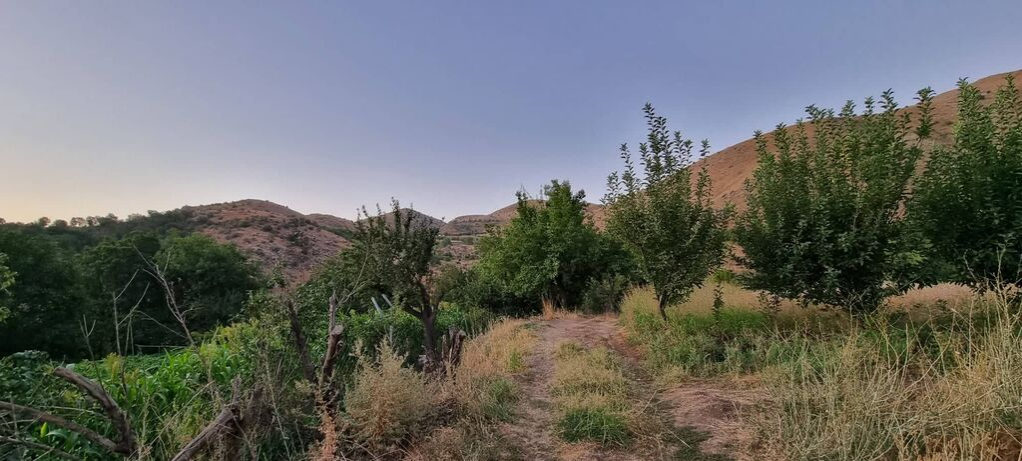
Collecting site in Amadiya (photograph by H. Ibrahim).

**Figure 5.**
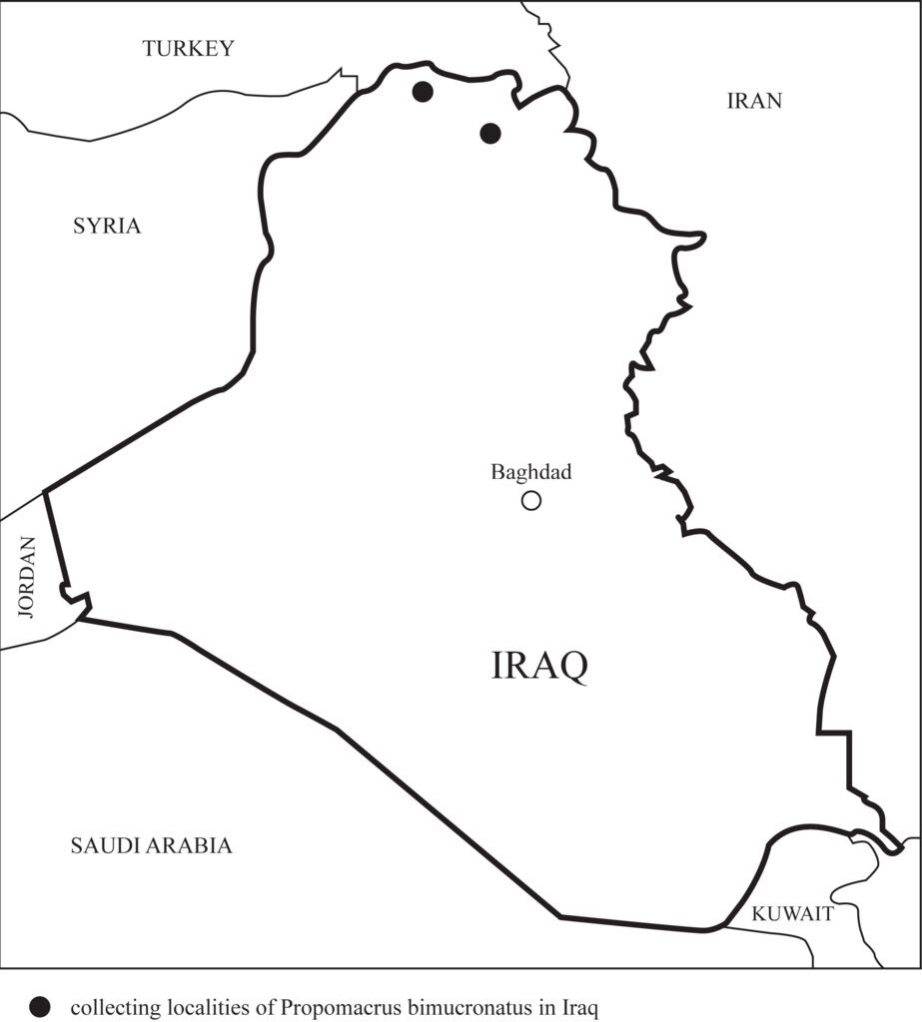
Map of Iraq with collecting localities of *P.bimucronatus* marked by black dots.

It is clear that the existence of *P.bimucronatus* depends entirely on the presence of old veteran trees with hollow trunks. The distribution of *P.bimucronatus* in Israel corresponds to the oldest and less-disturbed forests. The recent specimens, collected in 2022 in populated places in the Lower Galilee, were found close to relatively dense and old oak forests: Hosha'ya (Fig. 2) at the edge of Ya'ar Solelim Nature Reserve and Bet Lehem haGelilit at the edge of Allone Abba Nature Reserve. The beetles were most probably attracted to lights and arrived from the forest, although the specimen collected in Bet Lehem haGelilit was found in the yard near an oriental plane (not very large and not particularly old, with no signs of beetle activities, L. Friedman, pers. comm.).

We assume that *P.bimucronatus* is a semivoltine species, with adults appearing once in 2-3 years more or less simultaneously throughout their distributional range. However, this assumption needs to be proved by more observations in nature and study of additional material in the entomological collections. More intensive search for larvae in old rotten trees in Kurdistan is needed to reveal the host plants.

## Supplementary Material

XML Treatment for Propomacrus bimucronatus

## Figures and Tables

**Table 1.** The collecting localities of *Propomacrusbimucronatus* in Israel, according to the zoogeographical regions.

**Zoogeographical region of Israel**	**Locality**	**Date**	**Collector**	#
Har Hermon	Newe Ativ	26-29.viii.1981	A. Freidberg	2
Golan Heights	Panyas	30.vii.2002	V. Kravchenko, V. Chikatunov	1
Hula Valley	Dan	20.x.1966	Avshalom	1
Hula Valley	Dan	ix.1981	E. Horovitz	1
Hula Valley	Sede Nehemya [Huliyot]	27.viii.1973	Z. Shoham	1
Hula Valley	Sede Nehemya [Huliyot]	13.ix.1973	Z. Shoham	1
Hula Valley	Senir	22.viii.1969		1
Upper Galilee	Nahal Keziv	20.v.1999	V. Chikatunov	1
Upper Galilee	Nahal 'Ammud	23.ix.2003	V. Kravchenko, V. Chikatunov	1
Lower Galilee	Hosha'aya	9.vii.2022	R. Haritan	1
Lower Galilee	Bet Lehem haGelilit	12.viii.2022	O. Lior, G. Tamir, I. Barkai	1
Yizre'el Valley	Bet She'arim	16.ix.1957	A. Yoffe	1
Carmel Ridge	Haifa, Carmel	6.vii.1945	H. Bytinski-Salz	1
Carmel Ridge	Carmel Ridge, Horeshat haArba’im	10-31.vii.2009	J. Buse	1
Carmel Ridge	Carmel Ridge, Horeshat haArba’im	31.vii-13.viii.2009	J. Buse	1
Carmel Ridge	Carmel Ridge, Horeshat haArba’im	28.viii-10.ix.2009	J. Buse	4
Central Coastal Plain	Pardes-Hanna	vi.1946	H. Bytinski-Salz	1
Central Coastal Plain	Pardes-Hanna [Meged]	5.ix.1948	H. Bytinski-Salz	1
Central Coastal Plain	Tel Aviv	summer 1954		1
Southern Coastal Plain	Miqwe Yisrael Agricultural School	23.vii.1945		1
Foothills of Judea	Sha'ar haGay	iv.1958	M. Samish	1
Judean Hills	Yerushalayim, Refa’im Valley	11.ix.2014	A. Orlov	1
